# Mr. Ernest Hart

**Published:** 1898-03

**Authors:** 


### OBITUARY.

### Mr. Ernest Hart.

Ernest Hart, editor of the *British Medical Journal*, died in London on January 7th. He was born in June, 1836. He was educated at the City of London School and the School of Medicine attached to St. George's Hospital. At one time he was ophthalmic surgeon and lecturer on ophthalmology at St. Mary's Hospital. He rendered great service in exposing defective arrangements for sick poor in workhouses, his efforts leading to the passage of Hart's Act and the creation of the Metropolitan Asylums Board. His reports on criminal baby farming in 1868 led to the passing of the Infant Life Protection Act.

Mr. Hart was Honorary Chairman of the National Health

Society and President of the Medical Sickness, Annuity and Life Assurance Society. He was appointed editor of the *British Medical Journal* in 1866. and for several years was responsible for the editorial conduct of the *Sanitary Record* and the *London Medical Record.*

